# The Sheffield Medical School

**Published:** 1889-10-12

**Authors:** 


### THE SHEFFIELD MEDICAL SCHOOL.

The session was inaugurated by Mr. Charles Atkin, lecturer on anatomy. Here, again, the lecturer gave an epitome of the history of anatomy and surgery. And in common with the lecturers already referred to, Mr. Atkin lays much stress on the value of preliminary training of the student. At the same time the student must not neglect indulgence in exercise and sports. The subject of healthy homes was next discussed, and disease was stated to be often not only the result of drunkenness and immorality, but also of environment. With regard to hospitals, Mr. Atkin said that more care might be taken in checking the attendance of people who could well afford to pay a medical man a reasonable fee.

